# Influence of Socioeconomic Factors, Gender and Indigenous Status on Smoking in Taiwan

**DOI:** 10.3390/ijerph13111044

**Published:** 2016-10-25

**Authors:** Liang-Ting Tsai, Feng-En Lo, Chih-Chien Yang, Wen-Min Lo, Joseph Jordan Keller, Chiou-Wei Hwang, Ching-Feng Lin, Shu-Yu Lyu, Donald E. Morisky

**Affiliations:** 1 Taiwan Marine Education Center, National Taiwan Ocean University, Keelung City 20224, Taiwan; liangting.tsai@gmail.com; 2 Institute of Indigenous Health and Preventive Medicine Research Center, Taipei Medical University, Taipei 11031, Taiwan; e8504002@apc.gov.tw; 3 Department of Medical Research, China Medical University Hospital, China Medical University, Taichung 40402 Taiwan; lo.fengen@msa.hinet.net; 4 Department of Leisure and Recreation Management, Asia University, Taichung 41354, Taiwan; 5 Graduate Institute of Educational Measurement and Statistics, National Taichung University of Education, Taichung 40306, Taiwan; noahyang@mail.ntcu.edu.tw; 6 Department of Information Management, Tatung University, Taipei 10452, Taiwan; 7 School of Public Health, Taipei Medical University, Taipei 11031, Taiwan; joseph.jordan.keller@gmail.com (J.J.K.); m508102001@tmu.edu.tw (C.-W.H.); 8 Department of Leisure Industry and Health Promotion, National Taipei University of Nursing and Health Sciences, Taipei 11219, Taiwan; 9 Department of Community Health Sciences, Fielding School of Public Health, University of California at Los Angeles, Los Angeles, CA 90095, USA; dmorisky@ucla.edu

**Keywords:** indigenous, ethnic, gender, smoking, trends, tobacco tax increase, Taiwan

## Abstract

The indigenous Austronesian minority of Taiwan is heavily affected by health disparities which may include suffering from a greater burden of the tobacco epidemic. While a lack of representative data has historically precluded an investigation of the differences in smoking between Taiwanese ethnicities, these data have recently become available through an annual population-based telephone survey conducted by the Health Promotion Administration, Ministry of Health and Welfare (previously known as the Bureau of Health Promotion (BHP), Department of Health). We used the BHP monitoring data to observe the prevalence of smoking and environmental tobacco smoke exposure among indigenous and non-indigenous Taiwanese surrounding a tobacco welfare tax increase in 2006, investigate ethnic differences in smoking prevalence and environmental tobacco smoke exposure each year between 2005 and 2008, and perform multiple logistic regression to estimate measures of association between potential risk factors and smoking status. Despite significant ethnic and gender differences in smoking prevalence, smoking status was not found to be significantly associated with ethnicity after controlling for socioeconomic and demographic factors.

## 1. Introduction

Indigenous populations worldwide suffer from significant health disparities. American Indians/Alaskan Natives [1], the Maori of New Zealand [2], and the Aboriginals of Australia [3,4,5,6,7] all have poorer health than their non-indigenous counterparts. Indigenous populations moreover bear a disproportionate burden of substance-related morbidity and mortality when compared to non-Indigenous populations [8], including a heavier burden of the tobacco epidemic [7,8,9,10,11].

The American Indians and Alaska Natives of the United States [8], the First Nations of Canada [11], the Aboriginals and Torres Strait Islanders of Australia [9], and the Maori of New Zealand [10] all smoke more than any other racial or ethnic group in their respective countries. Smoking increases the short-term risks of adverse pregnancy outcomes [12], and the long-term risks of both neurodevelopmental disorders [13] and cancer [14]. Unfortunately, indigenous women in these countries experience a greater ethnic divide in smoking prevalence than men. Many smoke through pregnancy [9,15], and in some locations, smoke more than men [10,16], thus strongly contributing to both racial and gender health gaps.

The World Health Organization 2008 Report on the Global Tobacco Epidemic laid out its MPOWER policy package (Monitor, Protect, Offer, Warn, Enforce, Raise) enshrining the monitoring of tobacco use and prevention policies as one of the six control measures central to stemming the tide of the tobacco epidemic [17]. The information gathered by accurate monitoring is critical to planning ethnic and gender sensitive tobacco cessation interventions. Thus, it is especially important to monitor tobacco use and evaluate tobacco cessation interventions among indigenous peoples as they represent a culturally distinct high-risk subset of the population that may be best reached by means apart from the general population.

Until recently, representative samples of the Taiwanese indigenous population have been unavailable. However, one recent small-scaled study has suggested that 57.4% of indigenous Taiwanese are current smokers [18]. The Health Promotion Administration, Ministry of Health and Welfare (previously known as the Bureau of Health Promotion (BHP), Department of Health) conducts a nationwide annual telephone survey to monitor the smoking habits of the Taiwanese population, thus making a representative population-based investigation of both indigenous and non-indigenous Taiwanese smoking behavior feasible.

The “R” in MPOWER refers to raising taxes on tobacco to combat smoking. Reduced tobacco consumption following the promulgation of tobacco taxes has been reported by hundreds of studies conducted in countries across the income spectrum [19]. Thus, to curb tobacco consumption, the Taiwanese government began imposing a tobacco welfare tax in 2002. We are unfortunately unable to effectively appraise the utility of the 2002 tax as the BHP had not yet launched its population-based tobacco monitoring telephone survey at that time. However, a tax increase was levied in 2006 doubling the total excise (10 New Taiwan Dollar (NT$) = 0.311 USD) which is captured in the BHP data. Therefore, this study set out to use the BHP monitoring data to investigate ethnic- and gender-specific trends in smoking prevalence between 2005 and 2008, estimate measures of association between potential risk factors and smoking status, and explore the prevalence of smoking surrounding a tobacco welfare tax increase in 2006.

## 2. Experimental Section

### 2.1. Sample and Data Collection

All data were derived from the Bureau of Health Promotion (BHP) nationwide telephone surveys for the years 2005–2008. Every interviewer was professionally trained and conducted a standardized interview. Supervisors monitored the interviewers to make sure that they followed their script precisely, as well as to ensure interview uniformity. We used every smoking-related item from these surveys in our analysis. These surveys have been conducted annually in Taiwan beginning in 2004 and use the probability proportional to size technique to randomly sample a new cohort of Taiwanese adults aged 18 years and over annually. On average, the nationally-representative samples consisted of more than 15,000 respondents annually. To comply with the Joint Institutional Review Board of Taipei Medical University (JIRB), this study was restricted to the adult population of Taiwan which was defined by the JIRB as consisting of those over 20 years of age. Therefore, we only analyzed the data for Taiwanese over 20 years of age and utilized the appropriate sampling weights as included in the BHP adult tobacco use surveys. Thus, both the non-indigenous and indigenous Taiwanese samples analyzed in this study were representative of the overall population. This strategy used the data collected from the Taiwan 2000 census for standardization and weighting, including sex, age, educational attainment, and residential population. The study was conducted in accordance with the Declaration of Helsinki, and the protocol was approved by the Joint Institutional Review Board of Taipei Medical University (201309004).

### 2.2. Measures

This study investigated the factors that the BHP included in their tobacco use surveys between 2005 and 2008. How these factors were utilized to perform this study’s analysis is described below.

#### 2.2.1. Dependent Variables

Smoking behavior and environmental tobacco smoke exposure were used as dependent variables to estimate the prevalence of smoking and environmental tobacco smoke exposure for each year of the study. Smoking behavior was also used as the dependent variable in our multivariable logistic regression models. Respondents were grouped into never-smokers and current smokers according to their responses to questionnaire items regarding their smoking habits. Never-smokers were those who never smoked or had smoked less than 100 cigarettes in their lifetime; current smokers were those who smoked over 100 cigarettes in their lifetime and reported smoking in the month prior to the interview day [20].

Having smoked more than 100 cigarettes was categorized into “yes” and “no”. “Yes” was numerically coded as 2 and “no” as 1. Exposure to environmental tobacco smoke was defined as either having co-workers smoke at the respondents’ place of work, or anyone smoke in the respondents’ residence in the week preceding the interview. Exposure to environmental tobacco smoke at home was categorized into “yes” and “no”. “Yes” was numerically coded as 2 and “no” as 1.

#### 2.2.2. Independent Variables

Nearly every demographic and smoking-related item in the BHP survey apart from smoking behavior and exposure was included as an independent variable in this study. The BHP survey included items questioning respondents on their age, ethnicity, educational attainment, gender, occupational status, self-rated health, and environmental tobacco smoke exposure at home. Here, we present how each independent variable was categorized and the numeric coding used for each level of the variable. Age was broken into the following age groups: 20–29 years (1), 30–39 years (2), 40–49 years (3), 50–59 years (4), and 60 years and above (5). Educational attainment was categorized into junior high school and below (1), senior high school (2), and junior college or above (3). Gender was categorized into male (2) and female (1). Marital status was categorized into never married (3), divorced/widowed/separated (2), and married/cohabitation (1). Average household income was categorized as: less than NT$20,000 (1), NT$20,001–NT$40,000 (2), and NT$40,001 or more (3). Employment was categorized as: fulltime (1) or unemployed/part-time (2). Self-rated health status was categorized as: very poor/poor/fair (1) and very good/good (2). Ethnicity was categorized as indigene (1) and non-indigene (0).

### 2.3. Statistical Analyses

SPSS 18.0 statistical software (SPSS Inc., Chicago, IL, USA) was used to conduct all the statistical tests performed in this study. Descriptive statistics were provided to summarize the sample in terms of every item (demographic, socioeconomic, and smoking-related factors) included in the BHP telephone surveys. Chi-squared tests were used to evaluate differences between the indigenous and non-indigenous subjects in terms of the variables explored for each independent year of the study (2005–2008). We constructed one multivariable logistic regression model for each independent year of the study (2005–2008) by directly entering the independent variables taken from the BHP survey to test for associations between these independent variables and smoking status. The strength of association estimates were reported using odds ratios (OR) and 95% confidence intervals (CI). The statistical significance level was set at *p* values less than or equal to 0.05.

## 3. Results

Table 1 presents respondent socio-demographic characteristics and smoking related behaviors. Indigenous Taiwanese were more likely than non-indigenous Taiwanese to be current smokers in 2007 (31.2% vs. 23.5%, *p* = 0.002) and 2008 (27.3% vs. 23.2%, *p <* 0.001), although the difference in smoking between the ethnicities did not reach the significant level in either 2005 (28.6% vs. 24.1%, *p* = 0.096) or 2006 (26.6% vs. 23.4%, *p* = 0.194). Indigenous Taiwanese were also more likely to have smoked over 100 cigarettes in 2007 (38.4% vs. 30.9%, *p* = 0.008), but not any other year. Furthermore, environmental tobacco smoke exposure was found to be more common among indigenous Taiwanese than non-indigenous Taiwanese in 2005 (53.2% vs. 44.1%, *p* = 0.003), 2007 (42.9% vs. 30.9%, *p <* 0.001) and 2008 (37.5% vs. 27.4%, *p* = 0.012), but not 2006 (41.9% vs. 33.4%, *p* = 0.144).

Table 2 presents the results of four multiple logistic regression analysis models that were constructed to identify the statistically significant relationship between smoking and selected characteristics among all the participants across the four-year study period. Multiple logistic regression analyses indicated that age, gender, educational attainment, occupational status, self-rated health, and environmental tobacco smoke exposure were significantly related to smoking prevalence in 2005, 2006, 2007, and 2008. Ethnicity was not associated with smoking status in any year of the study after adjusting for other demographic and socioeconomic factors. While indigenous Taiwanese were not more likely to smoke simply based on their ethnic affiliation, men were far more likely to smoke than women simply based on their gender. This was true for 2005 (OR = 17.90, 95% CI = 15.40–20.80), 2006 (OR = 17.88, 95% CI = 15.30–20.80), 2007 (OR = 14.65, 95% CI =12.70–16.80), and 2008 (OR = 14.95, 95% CI = 13.00–17.10). Having never married was associated with a lower likelihood of smoking than having married in every year of the study period except for 2008, while being divorced, widowed, or separated was associated with a higher likelihood of smoking in every year of the study. Lower household incomes (<NT$20,000 and NT$20,001–NT$40,000) were associated with a lower likelihood of smoking than higher household incomes (>NT$40,001) in 2005, 2007, and 2008, while there was no statistically significant difference in smoking between income groups in 2006. The ORs of all variables can be seen in Table 2.

## 4. Discussion

This study detected significant differences in the overall prevalence of smoking across genders and ethnicities. Irrespective of ethnicity, men consistently smoked substantially more than women throughout the study period. Indigenous Taiwanese smoked significantly more than their non-indigenous counterparts throughout the latter half of the study. However, while differences in smoking prevalence did persist between genders and ethnicities, after adjusting for age, educational attainment, gender, marital status, average household income, occupational status, self-perceived health status, and exposure to environmental tobacco smoke at home, multiple logistic regression analysis revealed that ethnicity was not associated with smoking status.

Irrespective of ethnicity, male smoking in Taiwan dwarfs that of female smoking. This is in agreement with the global norm in which the percentage of male smokers far exceeds that of female smokers, with smaller gaps being observed in high-income countries [20]. The rate of non-indigenous Taiwanese female smoking was found to be low (4.7%–5.9%) when compared with Western countries such as the US (15.3% in 2015) [21], and are more similar to those reported by the World Health Organization (WHO) for Han Chinese (4% in 2008) [17]. On the other hand, the rates of male smoking in Taiwan (39.2%–40.4%) fall between higher rates in China (61% in 2008) [17] and lower rates in the US (20.5% in 2015) [21].

The smoking prevalence among indigenous Taiwanese was significantly higher than that of non-indigenous Taiwanese during the final two years of the study. This is in agreement with the data gathered from indigenous populations around the world: indigenous peoples bear a heavier burden of the tobacco epidemic. In the United States, 32.4% of the American Indians and Alaska Natives smoke, the highest prevalence of smoking by far among any racial or ethnic group [9]. Furthermore, in 2004 American Indian and Alaska Native youth (23.1%) had the greatest cigarette smoking prevalence among youth in the country, followed by non-Hispanic whites (14.9%), Hispanics (9.3%), non-Hispanic blacks (6.5%), and Asian Americans and Pacific Islanders (4.3%) [9]. The smoking prevalence among First Nations (59%) and Inuit (58%) in Canada is even greater, with the majority (52%) of First Nations starting between the ages of 13 and 16 and almost half (46%) of Inuit starting at age 14 or younger [11]. In Australia, 45% of Aboriginals and Torres Strait Islanders smoked in 2008, which is more than double the rate found among the overall Australian population (20%) [9]. In New Zealand, the smoking rate for Maori was 44% in 2009, which is also more than double that of non-Maori (18%) [10].

There is likely to be some degree of cultural component underlying the difference in these rates. Native Americans consider tobacco to be a sacred gift and use it during religious ceremonies and as traditional medicine [22], Aboriginals were paid with tobacco in the early days after the arrival of Europeans to Australia [23], and the First Nations specifically use tobacco to pray, give thanks to the Creator and Mother Earth, communicate with the spirits, and purify the mind and heal the body [11]. However, indigenous Taiwanese have a history apart from other indigenous communities, and the results of our regression analysis indicate that smoking in Taiwan is not significantly associated with ethnicity, but rather with socioeconomic and environmental factors. Nevertheless, even in the absence of ethnic effects, the indigenous population of Taiwan continues to share a greater burden of the tobacco epidemic than the non-indigenous population, thus contributing to the health gaps observed among the indigenous community globally.

A more specific and likely more deleterious aspect of indigenous smoking behavior is the high prevalence of female smoking found in this study and abroad. Despite indigenous men having the greatest smoking prevalence of any demographic group in 2006 and 2008, the gender disparity among indigenous Taiwanese was substantially smaller than that of non-indigenous Taiwanese on account of the comparatively larger number of indigenous women who smoke. Although drawing from disparate cultures, indigenous women globally have higher smoking rates than their non-indigenous counterparts. In Australia, 50% of Aboriginal mothers smoke through pregnancy [9]. While only 17.8% of American Indian and Alaska Native women were found to smoke through pregnancy in 2005, they had the highest rate in the US when compared with non-Hispanic white (13.9%) and non-Hispanic black (8.5%) women [15]. In Canada, the number of indigenous young women who smoke exceeds the number of indigenous young men [16], and by adulthood the proportion of daily smokers among women (42.8%) almost matches that of men (43.5%) [16]. However, among the Maori of New Zealand, the women (48.3%) in adulthood smoke substantially more than the men (39.3%) [10].

Maternal smoking, and exposure to environmental tobacco smoke, not only increases the risk of adverse pregnancy outcomes, such as spontaneous abortion, stillbirth, preterm birth, and fetal growth restriction [12], but can also have long-term effects, such as neurodevelopmental disorders [13] and cancers [14]. Moreover, women often play a vital role in child rearing, and in addition to exposing their child in utero, women who continue to smoke past pregnancy further expose their child to environmental tobacco smoke after birth. Thus, indigenous female smoking is a grave public health concern in both Taiwan and abroad as it likely strongly contributes to the health gaps observed between indigenous and non-indigenous populations.

In the US and EU, a 10% increase in the real price of cigarettes has been estimated to reduce smoking prevalence by 1%–2% and consumption by 2%–5% [24,25]. The welfare tax increase in 2006 represented about an 11.11% increase in the real price of the cheapest brands of cigarettes commonly sold in convenience stores and roadside stands where cigarettes are routinely purchased in Taiwan. The sole study in the literature investigating the effect of tobacco taxes on an indigenous population noted a 2.2% decrease in consumption across 18 stores in remote Australian aboriginal communities during the seven months preceding and following the imposition of a 25% tax excise increase [26].

While our data collection did not include any investigation of how the increase in tax affected the indigenous population beyond consumption alone, the prior study found that the study population demonstrated increased demand to share cigarettes and an increased reliance on those with more disposable income to purchase cigarettes. Those who did not quit or reduce their smoking cited their dependence, the normative nature of smoking, and the lack of support to quit as principal reasons [26]. The Taiwanese indigenous community has a strong cultural emphasis on “sharing”, and thus it is possible that the strong social capital which provides resilience for the community in times of scarcity may have actually militated against the welfare tax’s purpose of decreasing smoking rates. In addition to the more normative nature of smoking among men than women in Taiwanese indigenous culture, and thus a greater use of social capital to provide resilience against the increasing prices, another possible explanation stems from a study conducted in Britain which concluded that men are less responsive to changes in price, and more responsive to health publicity, thus suggesting the increased use of health publicity to persuade the male indigenous Taiwanese population [27].

Increases in tobacco taxes provide windows of opportunity in which many smokers attempt to quit, and even more consider quitting. One study assessing the effect of a 2009 federal tax increase in Minnesota reported that 65% of their sampled smokers thought about quitting at the time of the tax increase, and 29% made a quit attempt due to the tax increase [28]. Thus, while many smokers are spurred from precontemplation to contemplation, and even the action stages of the transtheoretical model, not nearly as many (1%–2%) successfully make it to the final maintenance stage.

The study conducted in Minnesota also noted that while the tax increase explained 36.1% of the variance in cessation hotline calls and 7.6% of the variance in tobacco cessation program enrollments, the effect of a simultaneously waged media campaign explained 34% of the variance in cessation hotline calls and 64% of the variance in tobacco cessation program enrollments [29]. Their results are supported by prior studies which noted the importance of the media in increasing hotline call volume [30,31,32,33]. The Minnesota study further noted that the combined effect of the tax and media only increased hotline call volumes and enrollments for a short period leading up to the promulgation of the tax, and the first six weeks following its imposition. This pattern has also been noted by another study [34] which only reported increases in call volumes for the two weeks prior and four weeks following a tobacco tax increase in Maine. While Taiwan has a tobacco cessation hotline and other tobacco cessation support services, including discounted fees for smoking cessation clinics, unfortunately, the increase in tobacco welfare tax was not timed to coincide with any increase in media promoting these services, a smoking cessation campaign, or the implementation of any other tobacco control policy. To make full use of the opportunity afforded by the tax to decrease smoking, future increases in the tobacco welfare tax should be timed in concert with a proactive media campaign alerting smokers to both the tax and the services available to aid in their cessation.

While this study was able to shed light on indigenous Taiwanese smoking by analyzing a rare representative sample, it is also vulnerable to a number of limitations which we were unable to redress. First, this study was based on data collected by a telephone survey, and while the vast majority of Taiwanese have telephones, it is still possible that some do not. Secondly, although there are no official statistics tracking indigenous residences, the authors’ experience leads them to believe that the indigenous population moves more often than the non-indigenous population. If this were the case, they may often switch phone numbers with an intervening “black out” period during which they may not be reached by telephone. Third, indigenous Taiwanese are more likely to engage in employment requiring them to work until later in the evening, may be less available to take a telephone call, and thus less likely to be interviewed. If the general population of indigenous Taiwanese is indeed difficult to reach by telephone, it is possible that the sample of indigenous Taiwanese analyzed in this study may differ from the general population of indigenous Taiwanese in ways that would make them more likely to be reached. This may include a more stable economic situation. Fourth, the indigenous population of Taiwan only composes 2% of the overall population. Therefore, while we did possess sufficient statistical power to perform the analyses conducted in this study, a larger sample would have allowed us to generate more specific measures of association. Fifth, as the data were collected by telephone interview it is possible that the study suffered from some degree of social desirability bias with regard to smoking habits. If one ethnicity experienced social desirability bias more than the other it is possible that this effect engendered a bias in our comparison between ethnicities. Finally, we were unable to assess the indigenous participation rates for this study and thus were also unable to ensure the representativeness of the cohorts analyzed.

## 5. Conclusions

In this study, smoking status was not found to be significantly associated with ethnicity, but rather socioeconomic and environmental factors. However, disparities in the burden of tobacco across both gender and ethnic lines continue to persist and need to be addressed to close prevailing gaps in health. It is the hope of the authors that the government is cognizant of these results and uses them to design and conduct gender-specific and both ethnically- and culturally-tailored smoking prevention initiatives that are designed to work in concert with increases in tobacco taxes.

## Figures and Tables

**Table 1:** Respondent sociodemographic characteristics and smoking related behaviors by ethnicity, 2005–2008.

Variable	2005 (*N* = 15,555)			2006 (*N* = 15,963)			2007 (*N* = 15,623)			2008 (*N* = 15,884)		
	Non-Indigene	Indigene	*p*	Non-Indigene	Indigene	*p*	Non-Indigene	Indigene	*p*	Non-Indigene	Indigene	*p*
	%	%		%	%		%	%		%	%	
Age			0.072			0.063			0.387			0.03
20–29	23.2	24.6		23	23.5		22.5	21.8		23	30	
30–39	24.9	25.4		24.7	27.5		24.9	25.5		24.8	228	
40–49	22.2	24.6		22.3	21.9		22.4	26.5		22.3	20.1	
50–59	12.7	14.9		13.2	16		13.1	12.3		13.2	10.7	
60 and above	17	10.5		16.8	11.1		17.1	13.9		16.7	16.4	
Level of education			<0.001			<0.001			<0.001			<0.001
Junior high school and below	45	63.3		44.4	66.7		43.8	63.6		44.3	58.3	
Senior high school	27.9	23.2		28.1	23.1		28.5	23.8		28.2	29.3	
Junior college or above	27.1	13.5		27.5	10.2		27.7	12.6		27.5	12.4	
Gender			0.389			0.005			0.011			0.463
Male	51.1	53.9		51.4	43.1		50.9	58.5		50.8	48.8	
Female	48.9	46.1		48.6	56.9		49.1	41.5		49.2	51.2	
Marital status			0.038			0.11			0.036			0.533
Married/living together with someone	73.1	66.2		67.8	66.7		66.3	59.9		65.8	65.2	
Never married	23.9	29.7		24.5	22.5		24.9	27.7		25.8	24.8	
Divorced/widowed/separated	3	4.1		7.7	10.8		8.8	12.4		8.4	10	
Average household income												
Less than NT$20,000	26.3	43.8	<0.001	23.6	35.2	<0.001	24.2	38.5	<0.001	25.3	39.7	<0.001
NT$20,001–NT$40,000	27.3	25		27.2	30.1		26.5	28.7		29.2	34.2	
NT$40,001 or more	46.4	31.2		49.2	34.7		49.3	32.8		45.5	26.2	
Having a job			0.899			0.815			0.762			0.553
Fulltime	58.5	59		60.3	59.6		60.4	61.4		62.2	60.6	
None or part-time	41.5	41		39.7	40.4		39.6	38.6		37.8	39.4	
Self-rated health status												
Very poor/poor/fair	16.7	30.4	<0.001	19.7	25.8	0.011	20.2	28.7	<0.001	19.2	22.8	0.089
Very good/good	83.3	70.6		80.3	74.5		79.8	71.3		80.8	77.2	
Exposure to environmental tobacco smoke at home	44.1	53.2	0.003	33.4	41.9	0.144	30.9	42.9	<0.001	27.4	37.5	0.012
Ever smoked more than 100 cigarettes	29.8	32.7	0.314	31.2	29.2	0.491	30.9	38.4	0.008	31.2	35.1	0.113
Being a current smoker	24.1	28.6	0.096	23.4	26.6	0.194	23.5	31.2	0.002	23.2	27.3	<0.001

**Table 2:** Multiple logistic regression of smoking correlates, 2005–2008.

Variable	2005		2006		2007		2008	
	OR (95% CI)	*p*	OR (95% CI)	*p*	OR (95% CI)	*p*	OR (95% CI)	*p*
Age	0.97 (0.97–0.98)	<0.001	0.97 (0.97–0.98)	<0.001	0.97 (0.97–0.98)	<0.001	0.97 (0.97–0.98)	<0.001
Ethnicity								
Indigene	1.03 (0.72–1.49)	0.839	1.41 (0.98–20.02)	0.057	0.82 (0.58–1.15)	0.255	0.96 (0.70–1.30)	0.809
Non-indigene	1		1		1		1	
Level of education								
Junior high school and below	3.57 (3.01–4.23)	<0.001	2.70 (2.27–3.20)	<0.001	2.56 (2.17–3.01)	<0.001	3.28 (2.81–3.83)	<0.001
Senior high school	2.78 (2.39–3.24)	<0.001	2.76 (2.38–3.20)	<0.001	2.28 (1.97–2.62)	<0.001	2.57 (2.25–2.94)	<0.001
Junior college or above	1		1		1			
Gender								
Male	17.90 (15.40–20.80)	<0.001	17.88 (15.30–20.80)	<0.001	14.65 (12.70–16.80)	<0.001	14.95 (13.00–17.10)	<0.001
Female	1		1		1			
Marital status								
Never married	0.73 (0.63–0.85)	<0.001	0.80 (0.69–0.93)	0.005	0.72 (0.62–0.84)	<0.001	0.92 (0.80–1.05)	0.256
Divorced/widowed/separated	2.66 (1.95–3.63)	<0.001	1.71 (1.39–2.13)	<0.001	2.02 (1.67–2.45)	<0.001	1.58 (1.29–1.92)	<0.001
Married/living together with someone	1		1		1			
Average household income								
Less than NT$20,000	0.61 (0.51–0.73)	<0.001	0.91 (0.76–1.10)	0.369	0.78 (0.65–0.93)	0.005	0.81 (0.70–0.94)	0.008
NT$20,001–NT$40,000	0.77 (0.67–0.88)	<0.001	0.90 (0.79–1.03)	0.151	0.82 (0.72–0.85)	0.003	0.84 (0.75–.95)	0.007
NT$40,001 or more	1		1		1			
Having a job								
Fulltime	1.24 (1.08–1.43)	0.002	1.28 (1.10–1.48)	<0.001	1.24 (1.08–1.41)	0.002	1.27 (1.12–1.43)	<0.001
None or part-time	1		1		1			
Self-related health status								
Very poor/poor/fair	2.00 (1.74–2.29)	<0.001	1.81 (1.58–2.07)	<0.001	1.81 (1.60–2.06)	<0.001	1.69 (1.50–1.90)	<0.001
Very good/good	1		1		1			
Exposure to environmental tobacco smoke at home								
Yes	2.54 (2.28–2.84)	<0.001	2.63 (2.35–2.93)	<0.001	2.78 (2.50–3.09)	<0.001	2.64 (2.39–2.92)	<0.001
No	1		1		1			
Cox & Snell *R* square	0.271		0.261		0.25		0.249	
Nagelkerke *R* square	0.401		0.39		0.371		0.373	
